# Oxygen Reperfusion Damage in an Insect

**DOI:** 10.1371/journal.pone.0001267

**Published:** 2007-12-05

**Authors:** John R. B. Lighton, Pablo E. Schilman

**Affiliations:** 1 Department of Biological Sciences, University of Nevada at Las Vegas, Las Vegas, Nevada, United States of America; 2 Sable Systems International, Las Vegas, Nevada, United States of America; 3 Division of Biological Sciences, University of California at San Diego, La Jolla, California, United States of America; Universidade de Brasília, Brazil

## Abstract

The deleterious effects of anoxia followed by reperfusion with oxygen in higher animals including mammals are well known. A convenient and genetically well characterized small-animal model that exhibits reproducible, quantifiable oxygen reperfusion damage is currently lacking. Here we describe the dynamics of whole-organism metabolic recovery from anoxia in an insect, *Drosophila melanogaster*, and report that damage caused by oxygen reperfusion can be quantified in a novel but straightforward way. We monitored CO_2_ emission (an index of mitochondrial activity) and water vapor output (an index of neuromuscular control of the spiracles, which are valves between the outside air and the insect's tracheal system) during entry into, and recovery from, rapid-onset anoxia exposure with durations ranging from 7.5 to 120 minutes. Anoxia caused a brief peak of CO_2_ output followed by knock-out. Mitochondrial respiration ceased and the spiracle constrictor muscles relaxed, but then re-contracted, presumably powered by anaerobic processes. Reperfusion to sustained normoxia caused a bimodal re-activation of mitochondrial respiration, and in the case of the spiracle constrictor muscles, slow inactivation followed by re-activation. After long anoxia durations, both the bimodality of mitochondrial reactivation and the recovery of spiracular control were impaired. Repeated reperfusion followed by episodes of anoxia depressed mitochondrial respiratory flux rates and damaged the integrity of the spiracular control system in a dose-dependent fashion. This is the first time that physiological evidence of oxygen reperfusion damage has been described in an insect or any invertebrate. We suggest that some of the traditional approaches of insect respiratory biology, such as quantifying respiratory water loss, may facilitate using *D. melanogaster* as a convenient, well-characterized experimental model for studying the underlying biology and mechanisms of ischemia and reperfusion damage and its possible mitigation.

## Introduction

Oxygen is essential for most multicellular life-forms. However, it can also be toxic due to its biotransformation into reactive oxygen species (ROS). For example, many turtles are able to hibernate underwater for months but with a potential danger of ROS overgeneration during resumption of breathing. This is analogous to the situation of oxidative stress in mammalian organs subject to ischemia and reperfusion. In other words, the well-known deadly effects of anoxia (e.g., caused by ischemia) in higher animals such as mammals are caused in most cases not by anoxia *per se* but by subsequent reperfusion with O_2_ [1]–[3]. As Joanisse and Storey state, “Damage resulting from oxidative stress, defined as any condition where the rate of ROS production surpasses the ability of antioxidant systems to buffer them, has been demonstrated under numerous conditions (notably ischaemia-reperfusion, iron-overload and increased oxidative metabolism such as during exhaustive exercise). All cellular components are susceptible to attack by ROS. Damage to proteins, DNA and lipids (more particularly to polyunsaturated fatty acids) may result in loss of function, conformational changes and the formation of cytotoxic low molecular mass breakdown products” [4]. The use of the fruit fly *Drosophila melanogaster* as a model organism for studying responses to hypoxia and anoxia has, however, concentrated on gene expression, rather than on whole-organism physiological responses (e.g., [5]–[8]). The short-term responses of the intact, functioning fly to anoxic stressors and the effects of reperfusion, in terms of non-invasively quantifiable parameters such as mitochondrial respiration and the integrity of respiratory control systems, have to our knowledge received no attention, although the long-term effects of hypoxia and hyperoxia are attracting interest [9].

Indeed, to our knowledge, strong physiological evidence for reperfusion damage has yet to be reported in any invertebrate. This is surprising because insects in particular are ideal models for studying the whole-organism physiological effects of hypoxia, anoxia and reperfusion. This is because their cells do not depend on a functioning heart and bloodstream for respiratory gas exchange. Instead, they are directly exposed to air via the tracheal system. Briefly, insects possess highly branched, gas-filled tubes called tracheae ([10] and references therein), which connect the tissues to the outside air through small closeable valves called spiracles. O_2_ and CO_2_ are delivered about 200,000- and 10,000-fold, respectively, more rapidly in tracheal air than in aqueous environments such as blood [10], [11]. This makes it possible to monitor the effect of stressors at the cellular and sub-cellular level, for example the mitochondria, in near-real time without requiring a functioning circulatory system.

The spiracles are sensitive to internal O_2_ levels via the CNS, and react to changes by dilating or constricting their open area to maintain an adequate tracheal O_2_ concentration [12]. Thus hypoxia and anoxia are useful tools for eliciting information on respiratory control mechanisms at the whole-organism level of integration (e.g. [13]–[17]). Spiracles are actively closed by constrictor muscles. The integrity of these muscles, and that of their CNS-mediated control mechanisms, are vitally important because the tracheae are largely or entirely saturated with water vapor. Thus, faulty spiracular control leads rapidly to death by dehydration [18]–[20]. Spiracular area and thus the state of the spiracle control mechanism is easily assayed in a flow-through respirometry system [21] by measuring water vapor flux rate, which acts as an index of spiracular area [15], [17], [22], [23].

Here, we describe for the first time in any invertebrate animal the effects of anoxia-administered with both single and repeated reperfusions of O_2_-on metabolic kinetics (assayed by mitochondrial CO_2_ output), and on organismal integrity (assayed in this case by water loss rate, which is an index of spiracular control, and thus of the functional integrity of the neuromuscular and respiratory systems).

## Results and Discussion

### Single reperfusion

The mean mass of our flies was 0.804±0.010 mg (N = 48, i.e. 8 male flies at each of 6 anoxia durations; 7.5, 15, 30, 60, 90 and 120 minutes at 25°C). Prior to exposure to anoxia, mean water loss rate (WLR) was 0.0617±0.0034 mg hr^−1^ and mean rate of CO_2_ production (VCO_2_) was 3.23±0.12 µl hr^−1^ (N = 48 for each; this N holds for all subsequent statistics unless otherwise noted). The flies were continuously active. CO_2_ output was continuous, through slightly open spiracles. *D. melanogaster* can constrict, but cannot completely close, their spiracles [24].

**Figure 1:**
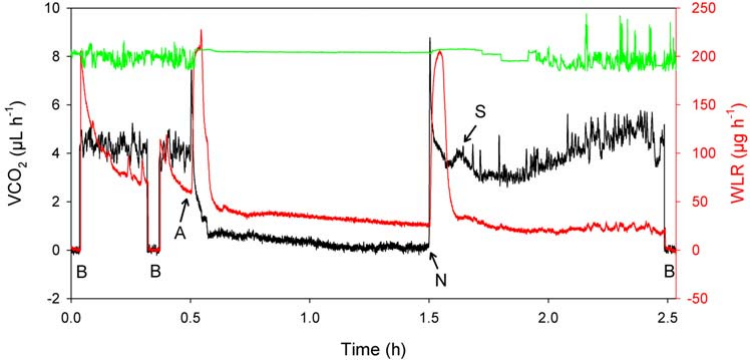
Entry into, and recovery from, a single bout of anoxia. Typical effects of 60 minutes of anoxia on the CO_2_ emission rate (VCO_2_; black), water loss rate (WLR; red) and activity (green; no units shown) of a male *Drosophila melanogaster*, mass 0.916 mg, at 25°C. B = baselines. A = initiation of anoxia. N = return to normoxia, S = secondary CO_2_ peak after reperfusion to normoxia. Note the increase in VCO_2_ after recovery from anoxia (recovery is evident in the activity trace).

A typical recording is shown in Fig. 1. Immediately after exposure to anoxia, the flies responded with a “spike” of CO_2_ output that reached a peak CO_2_ output rate, measured over the highest 10 seconds of the peak, of 6.77±0.21 µl hr^−1^. This is a highly significant increase over previous levels (*t* = 14.79, df = 94, P<10^−5^). Following this “spike”, CO_2_ output levels declined with first-order kinetics, reaching levels similar to baseline (zero) within 15 minutes.

It is to be expected that the fly's spiracles would open rapidly in anoxia, to maximize their open area [14], [15]. However, the CO_2_ “spike” preceded any increase in spiracular area, as measured by water vapor output. Therefore we presume that the “spike” may have originated from a rapid accumulation of intermediate acids such as lactate or acetate that drove buffered CO_2_ from the fly's hemolymph and tissues. Alternatively or in addition, it may have corresponded to increased activity levels (escape behavior) that took place when the fly first detected the drop in oxygen concentration. The lag-corrected water vapor signal peaked 101.2±7.4 seconds after the peak CO_2_ output, by which point all fly activity had ceased. This disparity was not caused by the relative positions of the analyzers in the gas path. The water vapor analyzer was placed before the CO_2_ analyzer, and the signals from both analyzers were lag-corrected in the analysis program to compensate precisely for the delays caused by their relative positions in the flow path. The response time of the water vapor analyzer was likewise not the cause of this effect, because excretory water loss signals showed a rise time approximately 5-fold faster than the signals originating from the opening of the spiracles. Because we know that the spiracles of *D. melanogaster* can react rapidly to changes in gas levels [15], we infer that this very slow spiracular response was caused by lack of O_2_. The highest sustained 10 seconds of WLR was 0.142±0.011 mg h^−1^, over double the pre-exposure levels (*t* = 7.07, P<10^−5^). It should be emphasized that the pre-exposure levels included both respiratory and cuticular WLR, whereas the anoxic increment was purely respiratory in origin, making this increase even more dramatic. With regard to WLR, it should also be noted that a decline over time in a dry atmosphere is normal in insects [25]. Thus, the pre-exposure estimate of WLR is likely an overestimate with reference to steady-state rates.

What happened next was unexpected. Instead of remaining open, the spiracles constricted again. Note that “constricted” does not equate to “closed”; as far as is known, *D. melanogaster* is unable to fully close its spiracles [24]. Overall WLR, measured over the most level 60 seconds occurring within the period of anoxia exposure, returned to low levels (0.0386±0.0035 mg hr^−1^). This WLR was significantly lower than the pre-anoxia rate (*t* = 4.67, P = 10^−5^), and indicates that the spiracle constrictor muscles were contracted to their maximum extent (though, as noted above, the spiracles could not be completely closed) in order to minimize WLRs. Respiratory gas exchange had, of course, ceased because of lack of oxygen. Because respiratory WLR in *D. melanogaster* is *ca.* 25% of total WLR [15], a 25% reduction from pre-exposure levels of 0.0617 mg hr^−1^ would be predicted (to 0.0463 mg hr^−1^). This does not differ significantly from observed levels (*t* = 1.52, P>0.1).

**Figure 2:**
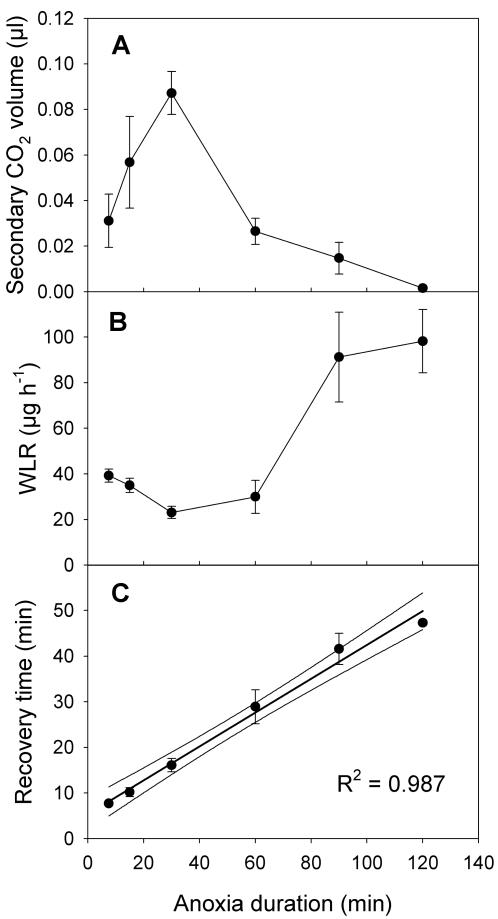
Effects of anoxia duration. The relation between anoxia duration and A) the volume of the secondary peak of CO_2_ emission after reperfusion to normoxia (measured by integration of the secondary peak against the background level of the primary peak, using sloping baselines). The secondary peak attains a maximum value after 30 minutes of anoxia exposure. Anoxia duration significantly affected the secondary peak areas (F_4, 32_ = 12.73, P<10^−6^); B) the minimum level of water loss rate (WLR) attained after reperfusion to normoxia, which is inversely related to the integrity of spiracular function. The longer the exposure to anoxia, the less adequately the spiracular control system recovered (F_5, 42_ = 9.94, P<10^−5^); C) the time required to resume voluntary activity (recovery). The longer the exposure to anoxia, the longer the time required for recovery. The dimensionless slope of the line is 0.399 (F_1,36_ = 169.0, P<10^−6^). Each point shown is the mean value for the number of flies (out of 8) that recovered; these are 8 for all anoxia durations except 90 minutes (5 recovered) and 120 minutes (only one recovered). Error bars are standard errors. The curves enclose the 95% confidence limits of the line fitted to data pooled by anoxia duration.

On reperfusion with O_2_, the first reaction was a large peak in CO_2_ output as the mitochondria were re-activated. The magnitude of this peak declined significantly with increasing duration of anoxia exposure (F_1, 46_ = 10.52, P = 0.002). This CO_2_ peak exited through spiracles that, although constricted, still allowed some gas exchange [24]; as noted above, the spiracles of *D. melanogaster* do not close completely, and so allowed the ingress of O_2_ and the egress of CO_2_. Following this “recovery spike”, a second, much smaller, peak of CO_2_ output was visible (see Fig. 1), the volume of which was far larger for intermediate durations of hypoxic exposure (15–30 minutes) than for longer or shorter durations (Fig. 2A). After 15 minutes of anoxia, the volume of the peak was inversely related to the duration of anoxic exposure (Fig. 2A). It is possible that this secondary peak is related to repair processes subsequent to reperfusion. In this respect it is suggestive that as the volume of this secondary peak declined with longer exposure to anoxia, recovery rates fell (see below).

Spiracular area, as traced by water vapor output, slowly increased after reperfusion began, and reached a maximum before returning again to lower levels. The time taken for water vapor output to reach peak levels, relative to the peak value of the CO_2_ “recovery spike”, was significantly affected by the duration of anoxic exposure. The longer the exposure to anoxia, the slower the response of the spiracles (F_1, 46_ = 228.8, P<10^−6^). Anoxia duration explains >83% of the variance in spiracle response delay. The 10-second peak value of WLR was not, however, affected by the duration of anoxic exposure, and was 0.1553±0.0122 mg hr^−1^. This did not differ significantly from the peak magnitude of the WLR peak that occurred after initial exposure to anoxia (*t* = 1.02, P>0.3).

The degree to which WLR (and thus spiracular area) returned to lower levels after reperfusion was highly significantly affected by the duration of anoxic exposure. We assessed the extent to which this recovery occurred by examining the WLR for 20 minutes after reperfusion, and finding the most stable reading over a three-minute window during that time. The stable reading occurred after the initial peak of WLR, and allowed us–by comparison with pre-anoxic WLR values–to assess the recovery of neuromuscular integrity following reperfusion. Longer durations of anoxic exposure caused incomplete spiracular recovery and thus higher post-reperfusion WLRs (Fig. 2B). However, this was not a simple linear relationship. As defined above, optimum recovery occurred after 30 minutes of anoxic exposure. Thus the best recovery from anoxia (closest return to pre-anoxia WLR, and thus spiracular control, levels) occurred following exposure to the intermediate anoxia durations that caused a distinct secondary CO_2_ emission peak (Fig. 2A), which we hypothesized might be repair-related.

Recovery of voluntary motor control following anoxia was assayed by the resumption of voluntary activity detected by the photoelectric activity sensor. The flies recovered voluntary motor control following anoxia after an interval of time that was linearly related to the duration of anoxic exposure (Fig. 2C). The slope of this relation, which is dimensionless and which we propose calling the anoxic recovery coefficient (ARC), was 0.399±0.031. This ARC is much steeper than reported elsewhere (0.14) over this range of anoxia durations [26]. This is presumably because our flies were exposed to practically immediate and complete hypoxia, rather than being subjected to prolonged first-order dilution washout in a container flushed with nitrogen.

All of the 8 flies exposed to anoxia for each duration up to and including 60 minutes recovered. Of the 8 flies exposed to 90 minutes of hypoxia, 5 recovered; of the 8 flies exposed to 120 minutes of anoxia, only one recovered.

### Multiple reperfusions

**Figure 3:**
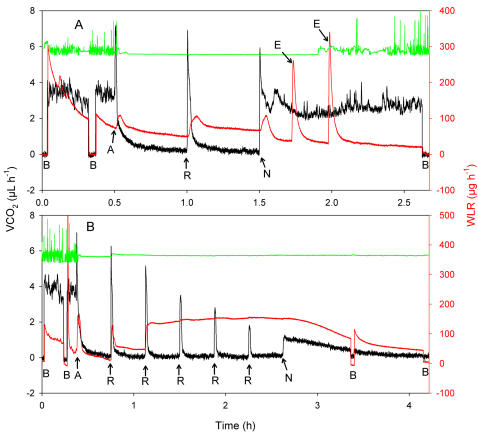
Effects of an oxygen reperfusion event followed by anoxia. Typical effects of 60 minutes of anoxia, interrupted by A) 1 minute of reperfusion with normoxia after 30 minutes, or B) multiple, 1 minute durations of reperfusion with normoxia, on the CO_2_ emission rate (VCO_2_; black), water loss rate (WLR; red) and activity (green; no units shown) of a male *Drosophila melanogaster* at 25°C. B = baselines. A = initiation of anoxia. R = reperfusion for 1 minute. N = return to normoxia. E = excretion events in the water vapor trace (red) after return to normoxia (approximately 1.75 and 2 hours); note the fast rise-times of these events.

A single, 60 second reperfusion to normoxia in the middle of a 60 minute exposure to anoxia caused a large “recovery spike” of CO_2_ output and a peak of WLR as the spiracles opened (Fig. 3A). Unlike the case with a single reperfusion to sustained normoxia after 30 minutes or an hour of anoxia, normal spiracular control did not resume after this brief reperfusion. Water loss rate increased from 0.0523±0.0089 mg hr^−1^ (N = 8 male *Drosophila*) prior to the brief reperfusion to 0.1535±0.0243 mg hr^−1^ after the post-reperfusion WLR peak. This nearly three-fold increase in WLRs is highly significant (*t* = 3.91, df = 14, P = 0.001). Even after the second, sustained reperfusion, WLRs did not return to normal levels indicative of full spiracular function. Instead, they declined to only 0.1159±0.0235 mg hr^−1^, significantly higher than rates recorded prior to the first reperfusion (*t* = 2.53, P = 0.025) and not significantly different from the elevated levels shown after the first reperfusion (*t* = 1.11, P = 0.28). Whereas all of the flies subjected to an hour of anoxia survived a single reperfusion to sustained normoxia, only 5 of the 8 flies exposed to the same duration of anoxia interrupted by a 60 second burst of reperfusion followed again by anoxia survived the return to sustained normoxia.

**Figure 4:**
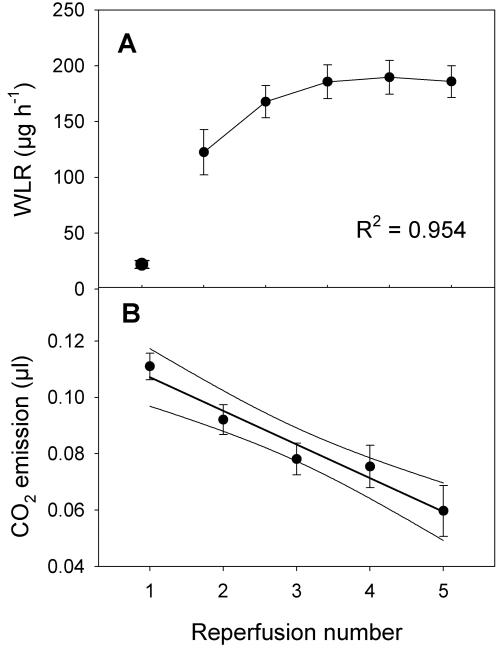
Effects of multiple oxygen reperfusions. The effect of successive reperfusions on A) water loss rate (WLR which is inversely related to spiracular control integrity). Successive reperfusions rapidly elevated water loss rates, and thus diminished spiracular control integrity (F_5, 30_ = 19.91, P<10^−6^). B) the volume of CO_2_ released by mitochondrial activity. Successive reperfusions rapidly reduced mitochondrial activity (F_5, 30_ = 19.91, P<10^−6^). Considered as a linear regression using the points shown, reperfusion number explained >95% of mitochondrial activity variance (F_1, 3_ = 62.0, P = 0.004). The curves enclose the 95% confidence limits of the fitted line. The five reperfusions lasted for 60 seconds each and were spaced 20 minutes apart. Each point shown is the mean value for 6 flies. Error bars are standard errors.

If a 60 second reperfusion was followed by a return to anoxia, as described above, and was then repeated multiple times at 20 minute intervals (Fig. 3B), the negative effect on spiracular function was cumulative and devastating (Fig. 4A). By the third reperfusion, WLR attained a sustained rate equivalent to the 10-second peak water output rate following a single, sustained reperfusion (0.1553±0.0122 mg hr^−1^). Meanwhile, the ability of the mitochondria to oxidize substrate declined highly significantly as the number of repeated reperfusions increased (Fig. 4B). Reperfusion injury is the parsimonious explanation for both effects. We assume that the repair of ROS-induced damage requires sustained reperfusion. Where reperfusion is intermittent and of short duration, ROS-induced damage that cannot be repaired in the absence of oxidative phosphorylation accumulates to an extent exceeding the ability of the flies to recover even when returned to continuous normoxia. It might be said that our technique “forces” reperfusion damage on an organism normally resistant to it. Obviously, quantitative determination of the ROS species involved, of the types of damage caused, and of the repair mechanisms impaired by repeated reperfusion, will be of considerable interest.

When using water vapor as a tracer gas for determining relative spiracular area, we are making the reasonable assumption that spiracular area is the primary determinant of variations in WLR (the cuticular component is continuous, and excretory events are easily recognized, e.g. Fig. 3A). We know that the spiracular area of *D. melanogaster* is variable and that this variability affects WLR [23] and, moreover, that spiracular area can respond rapidly to changes in external gas concentrations [15]. However, it is also possible that the water loss signals accompanying anoxic exposure and reperfusion may be due in part to modulations in the level of fluid in the tracheoles (the finely divided, distal branches of the tracheal system at which most gas exchange takes place, the surface area of which can be modulated by changes in fluid levels [27]). Direct observation of spiracular area may provide valuable additional information.

In summary, the deleterious effects of anoxia and reperfusion injury are important from both the pure and the applied (biomedical) perspectives. Much comparative work has been carried out on adaptations to life without oxygen; however, the mechanisms of reperfusion injury at the mitochondrial level are poorly understood. Here we employed some of the “classical” techniques of insect respiratory biology to explore and quantify the dynamics of recovery from anoxia and reperfusion in *Drosophila melanogaster*. The use of *Drosophila*, with its well-characterized genome, short generation time and ease of care and handling, as a model organism for furthering research on ischemia and anoxia holds promise. Using gas exchange parameters to assay mitochondrial recovery and the integrity of spiracular control is a simple, non-invasive procedure. We hope that it may assist in elucidating mechanisms and in evaluating or screening relevant strains and treatments in this important area of research.

## Methods

### Animals

We used male Oregon-R wild type Drosophila melanogaster between 5 and 15 days after eclosion for the measurements. They were reared and kept with media at a controlled temperature of 25±0.1°C.

### Respirometry

We used a sensitive flow-through respirometry system (SI-1/TR-2-SA system, Sable Systems International, Las Vegas, NV, USA [SSI]), which serves to minimize temporal errors [21] and allows the metabolic dynamics of the effects of anoxia, and subsequent recovery in normoxia, to be followed in real time in individual flies. We supplemented the system with a SSI RH-300 water vapor analyzer set to measure water vapor density in µg ml^−1^. Activity was monitored using LED and phototransistor probes. Specimen temperatures were controlled at 25±0.1°C with an SSI PELT-5 temperature cabinet. SSI's SSI UI-2 analog measurement interface and ExpeData software were used for data acquisition and analysis. Switching from normal air to pure nitrogen (N_2_), i.e., anoxia, utilized a pair of solenoid valves controlled by the data acquisition software and hardware (Figure S1). In their quiescent state, the solenoids allowed ambient air scrubbed of water vapor and CO_2_ to be pulled through the respirometry system at a rate of 50 ml min^−1^. Energizing the solenoids (response time<50 milliseconds) caused the system to pull N_2_, which flowed into a manifold at 250 ml min^−1^, from that manifold instead. The N_2 _manifold eliminated pressure changes and allowed N_2_ to be pulled through the system at precisely the same flow rate as the normoxic air scrubbed of water vapor and CO_2_.

The changeover from air to N_2_ took place in less than 10 seconds as measured with a SSI PA-10 paramagnetic O_2_ analyzer with a response time of <0.5 second. Very small CO_2_ and water vapor scrubbers (<2 ml internal volume) downstream from the solenoid assembly prevented any disturbance of the baseline water vapor or CO_2_ entering the fly chamber (volume<1.5 ml) during the transition. Baselines were taken automatically by the data acquisition system. We could program the system to give any desired exposure to anoxia and to take multiple baselines across long recordings to compensate for analyzer drift. The latter is crucial for long duration recordings measuring small CO_2_ and H_2_O concentration increments from tiny organisms such as individual *Drosophila*, which typically yield a CO_2_ signal of <1 ppm at these temperatures and flow rates.

Bev-A-Line low-permeability tubing (Thermoplastic Processes Inc., Georgetown, DE, USA) was used throughout to minimize water vapor and CO_2_ absorbance errors. The chamber was a 6 mm i.d. polished bore through a 2.5 cm diameter×8 cm hexagonal nickel-plated aluminum rod, sealed with two thermally conductive sapphire windows (SSI isothermal *Drosophila* respirometry chamber). The polished bore allowed photoelectric detection through the sapphire windows of any movement by a single fly anywhere in the chamber. Two ports allowed passage of H_2_O-free and CO_2_-free air which was thermally equilibrated with the temperature of the cabinet by traveling through a 2 mm i.d. serpentine path milled through the length of the aluminum stock [17]. Finally, air left the respirometry chamber (having gathered CO_2_ and H_2_O from the fly on its way), entered the RH-300 water vapor analyzer and traveled to the CO_2_ analyzer (see Figure S1).

### Procedure

During a typical run, an individual male *Drosophila* was aspirated from the breeding container and placed in the chamber. The fly was minimally handled; no thermal or chemical tranquilization was applied. The recording began with a baseline segment to establish the zero points for the CO_2_ and water vapor analyzers. After that the CO_2_ and H_2_O released by the fly were measured for 24 minutes (plus another baseline). The incurrent air was changed to pure nitrogen (N_2_) for 7.5, 15, 30, 60, 90 or 120 minutes. For the 7.5 to 60 minutes of anoxia treatments, another 30 to 60 minutes (depending on the anoxia duration) was measured in normal air in order to determine recovery time, and the end baseline taken. For the longer runs, the recovery period lasted 100 or 180 minutes and both an intermediate and an end baseline was recorded.

In the case of reperfusions to brief normoxia, air was switched through the chamber for 60 seconds and then the system was returned to anoxia. In some runs (see below) the 60 second normoxic reperfusion was repeated at 20 minute intervals.

When the recording was complete, the fly was removed from the chamber and weighed to the nearest 1 µg using a Cahn C-32 ultramicrobalance (Cahn Instruments Inc., Cerritos, California). The mass of water lost during the recording, as determined from the respirometry data, was added to its post-recording weight [17]. Body mass and other relevant information were noted in the remarks of the saved file. Data were sampled at 1 Hz, using intra-sample finite impulse response digital filtration to reduce analyzer noise [21].

### Data analysis

Recordings were analyzed using ExpeData software. For each recording, the CO_2_ and H_2_O baselines were subtracted assuming linear or, where necessary, curvilinear drift. CO_2_ in ppm was converted to µl h^−1^ and H_2_O vapor density in µg ml^−1^ was converted to WLR in mg h^−1^ (see [21] for formulae). Because the CO_2_ and H_2_O analyzers were plumbed sequentially, the lag in response times for those traces was corrected individually. Cabinet and ambient temperature and air flow rate during the recording were also recorded.

Statistical summaries of selected sections of the recordings (means, etc.) were written to ExpeData's RudeStat spreadsheet where summary statistics were calculated and statistical tests were performed. Means are accompanied by SE (standard error of the mean) and N (sample size), and are compared using analysis of variance (ANOVA; >2 cases) or Student's *t*-test (2 cases). Regressions were by least squares, with significance testing by analysis of variance.

## Supporting Information

Figure S1 Not to scale. Nitrogen flow is adjusted to 250 ml/min when the solenoids are energized. The pump and flow meter (PUMP & FM) are set to 50 ml/minute. R = rotameter. NC = not connected. RC = Respirometry chamber. AMPC = Ascarite & magnesium perchlorate scrubber for removing CO_2_ and H_2_O. ADE = activity detector's emitter. ADD = activity detector's detector. H_2_O ANALYZER = water vapor analyzer. CO_2_ ANALYZER = infrared CO_2_ analyzer. Temperature controlled cabinet and controller, as well as electrical connections not shown for simplification. See text for details.
(0.37 MB TIF)
