# Supplementary figures and images for: Oxygen Reperfusion Damage in an Insect
Source: PLoS One. 2007 Dec 5;2(12):e1267. doi: 10.1371/journal.pone.0001267 (PMC2092388; doi:10.1371/journal.pone.0001267)

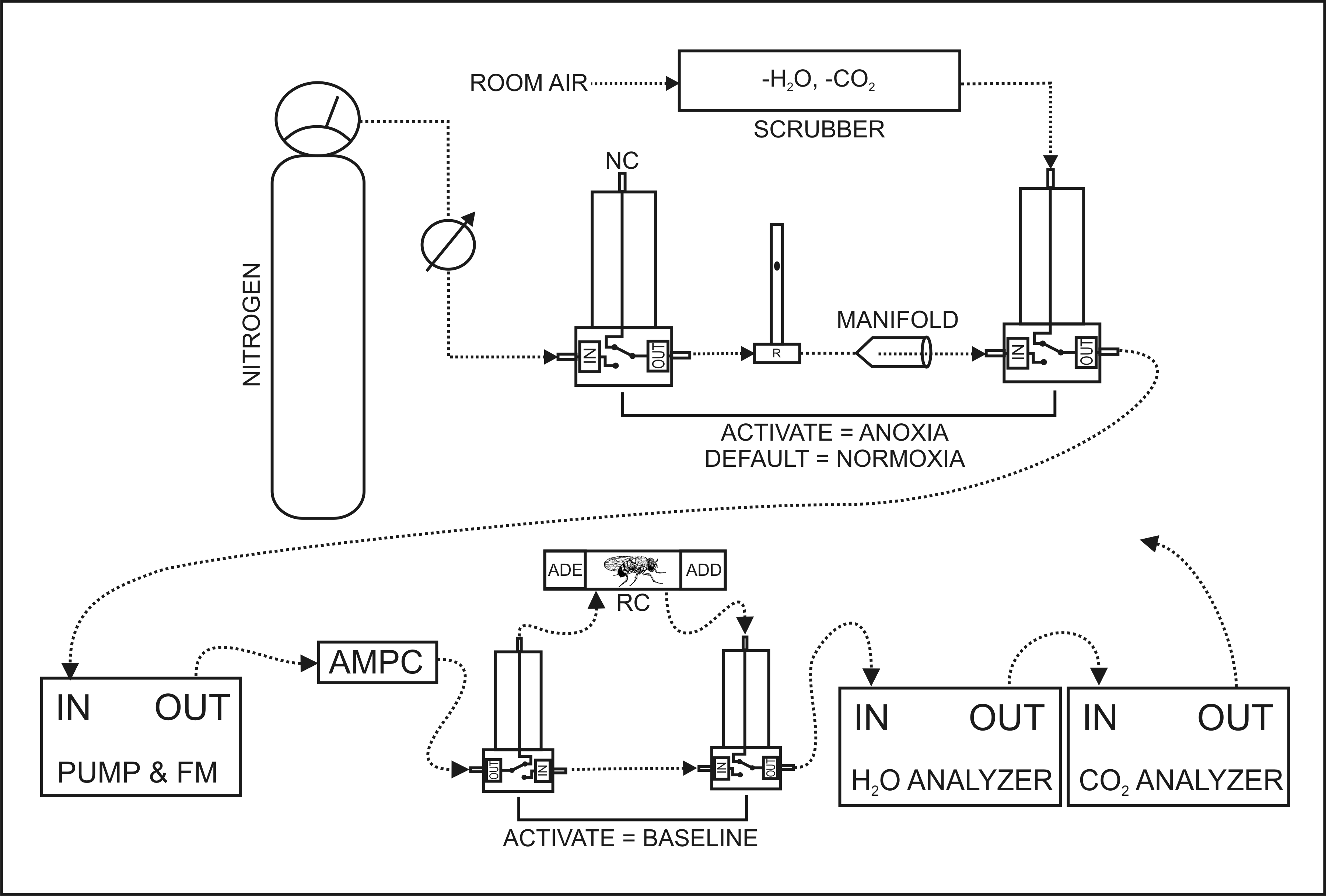

Supplement: Figure S1 — Not to scale. Nitrogen flow is adjusted to 250 ml/min when the solenoids are energized. The pump and flow meter (PUMP & FM) are set to 50 ml/minute. R = rotameter. NC = not connected. RC = Respirometry chamber. AMPC = Ascarite & magnesium perchlorate scrubber for removing CO2 and H2O. ADE = activity detector's emitter. ADD = activity detector's detector. H2O ANALYZER = water vapor analyzer. CO2 ANALYZER = infrared CO2 analyzer. Temperature controlled cabinet and controller, as well as electrical connections not shown for simplification. See text for details. (0.37 MB TIF) [file pone.0001267.s001.tif]
